# A Case of T1bN0 Esophageal Squamous Cell Carcinoma That Developed Recurrence 7 Years after Curative Esophagectomy

**DOI:** 10.70352/scrj.cr.25-0217

**Published:** 2025-09-17

**Authors:** Shinichiro Shiomi, Takashi Fukuda, Shintaro Nozu, Daiji Oka, Yusuke Yoda, Noriko Motoi, Hiroaki Kanda

**Affiliations:** 1 Department of Gastroenterological Surgery, Saitama Cancer Center Hospital, Kitaadachi-gun, Saitama, Japan; 2 Department of Gastroenterological Endoscopy, Saitama Cancer Center Hospital, Kitaadachi-gun, Saitama, Japan; 3 Department of Pathology, Saitama Cancer Center Hospital, Kitaadachi-gun, Saitama, Japan

**Keywords:** esophageal squamous cell carcinoma, late recurrence, lymphatic and vascular involvement, serum p53 antibody

## Abstract

**INTRODUCTION:**

The optimal follow-up period and appropriate examinations for patients with esophageal squamous cell carcinoma (ESCC) who have remained disease-free for 5 years remain controversial.

**CASE PRESENTATION:**

A 73-year-old man was diagnosed with ESCC and underwent curative esophagectomy. Pathological examination revealed a superficial tumor without lymph node metastases, despite lymphatic and vascular involvement. The patient underwent routine postoperative follow-up at our institution and showed no signs of recurrence until the 5th postoperative year. However, his serum p53 antibody titer increased 5 years postoperatively, and careful follow-up using imaging modalities, including CT, was scheduled. No lesions suspected of recurrence were noted over the next 2 years until bilateral pleural effusion was detected on CT in the 7th postoperative year. Cytological examination of the pleural effusion revealed pleural-seeded ESCC cells.

**CONCLUSIONS:**

Although several cases of late recurrence (>5 years) have been previously reported, most have a deeper infiltration depth than that of T2 or pathologically positive lymph nodes. However, patients with lymphatic/vascular involvement, even those with pT1bN0 ESCC, require careful surveillance using imaging modalities and laboratory tests, given the possibility of late recurrence occurring beyond 5 years.

## Abbreviations

ESCC
: esophageal squamous cell carcinoma
FDG-PET
: ^18^F-fluorodeoxyglucose PET

## INTRODUCTION

Approximately 80% of esophageal squamous cell carcinoma (ESCC) recurrences occur within 2 years after primary resection.^1)^ Although rare, several cases of late recurrence occurring after >5 years postoperatively, generally considered a cured period, have been reported.^2–10)^ Therefore, some patients must be followed up for >5 years postoperatively. However, the duration of postoperative outpatient follow-up and the decision to perform examinations vary among institutions and are inconsistent. Herein, we report a rare case of T1bN0 ESCC that recurred in multiple mediastinal lymph nodes and the pleura 7 years after curative surgery.

## CASE PRESENTATION

A 73-year-old man with no remarkable medical history except for a previous myocardial infarction was diagnosed with an esophageal lesion on esophagogastroscopy by a primary care physician and visited our facility for further examination. He consumed alcohol daily and smoked 15 cigarettes per day for 40 years. Laboratory tests revealed no remarkable abnormalities or tumor marker elevations.

**Fig. 1:**
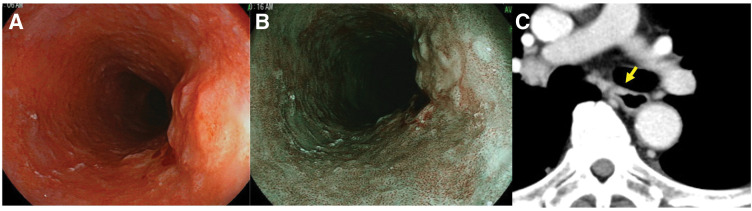
Images of esophagogastroscopy (**A**: white light; **B**: narrow band imaging) and chest CT scans (**C**) show a 0–IIc+0–Is lesion mainly occupying the right wall of the middle thoracic esophagus. The yellow arrows indicate the area of wall thickening on CT (**C**).

Esophagogastroscopy revealed a type 0–IIc+0–Is lesion, measuring 5 cm in length, located in the mid-thoracic esophagus and appearing to invade the submucosal layer (**Fig. 1A**, **1B**). Pathological examination of the biopsy specimen revealed squamous cell carcinoma. CT revealed wall thickening of the mid-thoracic esophagus (**Fig. 1C**). No findings suggested distant organ metastases or lymph node involvement. The patient was diagnosed with thoracic ESCC classified as clinical stage I (cT1bN0M0), according to the 8th edition of the American Joint Committee on Cancer/Union for International Cancer Control classification.^11)^

**Fig. 2:**
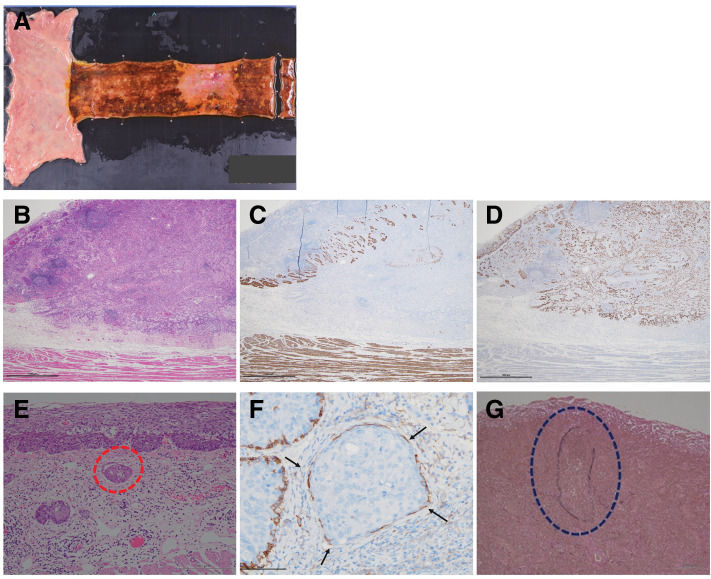
(**A**) Opened specimens of the esophagus and stomach show a 0–IIc+0–Is tumor in the middle thoracic esophagus. (**B**–**D**) Serial sections from the deepest portion of the tumor: (**B**) HE staining (×20), (**C**) immunohistochemical staining for desmin (×20), and (**D**) immunohistochemical staining for p53 (×20). (**E**–**G**) Histopathologic examination shows the lymphatic invasion: (**E**) HE staining (×100), (**F**) immunohistochemical staining of D2-40 (×200), and the vascular invasion: (**G**) Elastica van Gieson stain (×100). HE, hematoxylin and eosin

We performed a subtotal esophagectomy with 3-field lymphadenectomy using a combination of video-assisted transthoracic and hand-assisted laparoscopic approaches. Reconstruction was performed using a gastric conduit. The patient did not develop any postoperative complications and was discharged on the 27th POD. Histological examination of the resected specimen revealed moderately differentiated squamous cell carcinoma (**Fig. 2A**). The tumor cells invaded beyond the muscularis mucosa, as indicated by desmin staining, and the depth of pathological infiltration was diagnosed as pT1b (SM3) (**Fig. 2B**, **2C**). Immunohistochemical staining showed strong nuclear expression of p53 in the tumor cells (**Fig. 2D**). Among the 68 resected lymph nodes, no pathological metastasis was identified. Immunohistochemistry for AE1/AE3 was additionally performed on lymph nodes larger than 5 mm, which also showed no metastatic involvement. However, mild lymphatic invasion (ly1) and moderate vascular invasion (v2) were detected (**Fig. 2E**–**2G**).

**Fig. 3:**
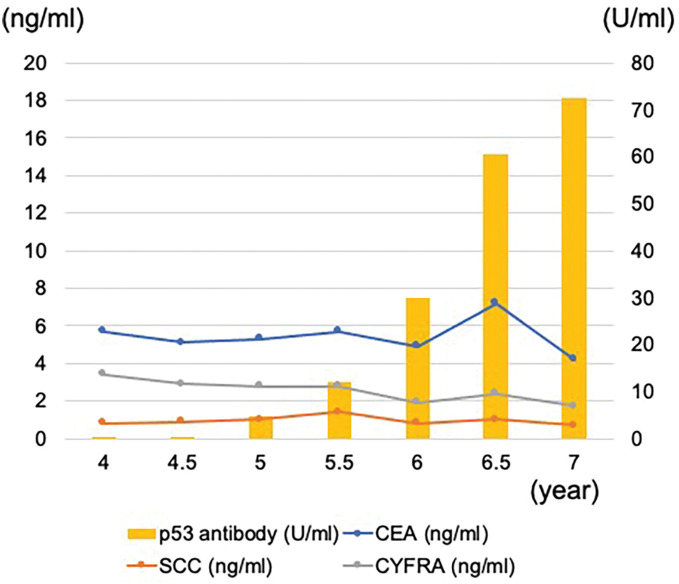
Time-dependent changes in tumor marker values beyond >5 years postoperatively. CEA, carcinoembryonic antigen; CYFRA, cytokeratin fragment; SCC, squamous cell carcinoma

Routine outpatient examinations for postoperative follow-up at our institution include laboratory tests and CT scans every 3 months for the first 3 years postoperatively and every 6 months thereafter. Esophagogastroscopy is performed annually. We generally continue this surveillance protocol for 8 years postoperatively in patients with pathological stage 0 or I cancer treated with surgery alone, and for 10 years in patients with stage II or higher disease or those who received neoadjuvant and/or adjuvant chemotherapy. The patient showed no signs suggestive of recurrence within the 5-year period. However, the serum p53 antibody titer increased above the normal limit (4.63 U/mL) in the 5th postoperative year and continued to rise thereafter. In contrast, other tumor markers (e.g., squamous cell carcinoma antigen, cytokeratin fragment, and carcinoembryonic antigen) remained within normal limits during this period (**Fig. 3**). The patient underwent further investigations, including CT, ^18^F-fluorodeoxyglucose PET (FDG-PET)/CT, colonoscopy, and bronchoscopy with bronchoalveolar lavage. No recurrence of esophageal cancer or other primary cancers was observed within 6.5 years after surgery.

**Fig. 4:**
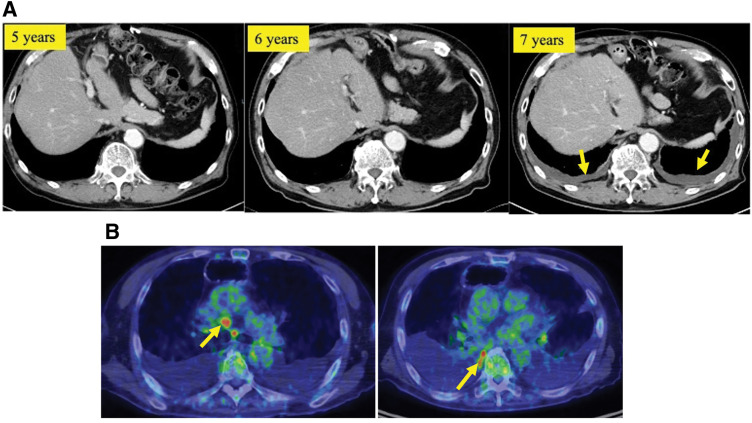
(**A**) Chest CT scan shows no appearance suspected of recurrence of esophageal squamous cell carcinoma until 6 years postoperatively. However, bilateral pleural effusions appeared on the CT scan conducted during the 7th postoperative year. (**B**) ^18^F-fluorodeoxyglucose PET/CT was conducted 3 months after the last CT scan and confirmed recurrence in multiple mediastinal lymph nodes and the right lateral pleura. The yellow arrows indicate bilateral pleural effusions on CT (**A**) and abnormal FDG uptake in mediastinal lymph nodes and the right pleura on PET/CT (**B**).

Nevertheless, a CT scan performed 7 years postoperatively revealed mild bilateral pleural effusion (**Fig. 4A**). Furthermore, an increasing trend of pleural effusion was suspected based on chest radiography performed 3 months later. FDG-PET/CT showed abnormal FDG uptake in multiple mediastinal lymph nodes and the right lateral pleura (**Fig. 4B**). Cytological analysis of the right pleural effusion obtained via percutaneous puncture revealed pleural-seeded squamous cell carcinoma cells. None of the CT or FDG-PET/CT scans performed during the postoperative follow-up period revealed findings suggestive of a new primary tumor in other organs. Based on the clinical course, the patient was diagnosed with ESCC recurrence in the pleura and multiple lymph nodes. Although systemic chemotherapy was considered, because the patient had developed dementia, he eventually decided to receive the best supportive care.

## DISCUSSION

Herein, we report a case of ESCC recurrence in multiple mediastinal lymph nodes and the pleura 7 years after curative esophagectomy for T1bN0 ESCC. **Table 1** summarizes the recurrence patterns and demographic characteristics of patients previously reported to have late ESCC recurrence. The most frequent site of recurrence was the mediastinal lymph nodes, followed by the cervical lymph nodes and lungs. Among patients reported with late recurrence, the median follow-up time until detection of the recurrent lesion was 7 years (range: 5–15 years). Two patients, reported by Miyamoto et al. and Hayashi et al., experienced recurrence more than 10 years after curative surgery.^4,5)^

**Table 1:** Previous case reports regarding late recurrence occurring beyond >5 years postoperatively

	Patient	Disease-free period	Recurrence site	Main lesion	TNM/stage (UICC 8th)	Procedure	Lymphadenectomy	Lymphatic/vascular invasion	Preoperative therapy	Postoperative therapy	Histological appearance
Uemura et al.^2)^	71F	7 years	Lung	Mt	pT3N1M0	TTE	3FD	No data	None	Chemotherapy	SCC
Sekiguchi et al.^3)^	68F	7 years	Lung	Lt	pT3N0M0	TTE	3FD	ly1, v1	None	Chemotherapy	Basaloid
Miyamoto et al.^4)^	79M	15 years	Cervical LN	Mt	pT3N1M0	TTE	3FD	ly1, v0	None	None	SCC
Hayashi et al.^5)^	59M	13 years	Mediastinal LN	Mt	pT3N0M0	TTE	3FD	No data	Chemoradiotherapy	Chemotherapy	SCC
Hayashi et al.^5)^	57M	6 years	Mediastinal LN	Mt	pT1N0M0	THE	3FD	ly0, v0	None	None	SCC
Hayashi et al.^5)^	62M	6 years	Mediastinal LN	Mt	pT3N1M0	TTE	3FD	No data	None	None	SCC
Ota et al.^6)^	77F	8 years	Abdominal LN	Mt	pT2N2M0	TTE	2FD	ly2, v0	None	Chemotherapy	SCC
Yamamoto et.al^7)^	66F	5 years	Cervical LN	Mt	pT2N2M0	TTE	2FD	ly1, v0	None	None	SCC
Nakano et al.^8)^	75M	9 years	Gastric tube	Ae	pT3N1M0	TTE	2FD	ly2, v1	None	Chemotherapy	SCC
Takahashi et al.^9)^	72F	5 years	Ovary	Lt	pT3N2M0	TTE	2FD	ly1, v1	None	None	SCC
Mori et al.^10)^	70M	9 years	Mediastinal LN	Lt/Mt	pT2N1M0	TTE	2FD	ly0, v0	None	None	SCC

Ae, abdominal esophagus; F, female; FD, field dissection; LN, lymph node; Lt, lower thoracic esophagus; M, male; Mt, middle thoracic esophagus; SCC, squamous cell carcinoma; THE, transhiatal esophagectomy; TTE, transthoracic esophagectomy; UICC 8th, the 8th edition of the UICC TNM classification

In a nationwide survey of follow-up practices for esophageal cancer conducted in 2020, more than 80% of certified institutions approved by the Japan Esophageal Society continued outpatient clinical visits for patients with stage 0/I diseases, even if they were recurrence-free 5 years after esophagectomy; however, approximately one-third of these institutions did not schedule CT scans beyond the 6th postoperative year.^12)^ The current case highlights the necessity of continuing careful outpatient follow-up for patients diagnosed with pT1bN0 ESCC.

Previous studies have reported that pathologically positive lymph nodes and advanced infiltration depth are possible risk factors for early and late postoperative ESCC recurrence.^13,14)^ As shown in **Table 1**, cases previously reported as the late recurrence of ESCC have pN ≥1 or pT ≥2 diseases, except for 1 case reported by Hayashi et al.^5)^. However, as the patient underwent transhiatal esophagectomy, the quality of mediastinal lymph node retrieval may have been reduced compared with that of conventional transthoracic esophagectomy. In contrast, the current patient was diagnosed with pT1bN0 disease despite a thorough mediastinal lymphadenectomy using the video-assisted transthoracic approach.

The patient had lymphatic and vascular invasion despite the absence of metastatic lymph nodes. Several previous studies have reported that lymphatic and/or vascular invasion is a risk factor for recurrence in patients without lymph node involvement.^15–17)^ Furthermore, lymphovascular invasion implies that tumor cells may infiltrate surrounding tissues and circulate systemically.^18)^ In the present case, unresected tumor cells may have remained after esophagectomy, although some inherent physiological mechanisms may have suppressed them during the long postoperative period.

Cancer dormancy, a state in which cancer cells exhibit a growth plateau below the detection level due to a dynamic equilibrium between cell proliferation and apoptosis, may account for recurrence following long-term disease-free survival.^19)^ Two mechanisms of cancer dormancy have been proposed: angiogenic dormancy, which involves a shortage of energy resources to cancer cells due to limited vascularization and angiogenesis, and immune-related dormancy, which is mediated by host CD8^+^ T cells.^20)^ However, the factors that disrupt the equilibrium between proliferation and apoptosis of tumor cells have yet to be elucidated.

Serum p53 antibody titer has been proposed as a tumor marker for predicting advanced tumor stages and poor prognosis in ESCC preoperatively.^21)^ Additionally, a post-therapeutic increase in serum p53 antibodies has been reported to be related to an unfavorable prognosis.^22)^ Although the preoperative p53 antibody titer was negative in the current case, it increased after a 5-year disease-free period. Previous studies have reported that p53 is involved in the differentiation and maintenance of different T cell types, including CD8^+^ and regulatory T cells.^23,24)^ Despite close follow-up with CT, recurrence was not diagnosed until 7 years postoperatively. However, elevated levels of the p53 antibody might reflect a failure of cancer dormancy, indicating the necessity of assessing late recurrence that could occur afterward. Hence, a time-dependent change in the p53 antibody titer may help detect recurrence at an early stage.

For patients diagnosed with pT1bN0 ESCC who have a disease-free period of 5 years, the optimal duration of follow-up and the most appropriate examinations thereafter have not been established. Several certified institutions reportedly discontinue clinical visits or follow-up imaging examinations beyond 5 years.^12)^ As noted above, we have maintained postoperative follow-up for up to 8 years, even in patients pathologically diagnosed with stage 0/I disease. This surveillance period appears appropriate for detecting late recurrence beyond 5 years after surgery in patients with pT1bN0 ESCC, as illustrated by the present case. In particular, patients with lymphatic or vascular invasion may require ongoing careful surveillance given the potential risk of late recurrence. Furthermore, our case highlights the potential value of monitoring p53 antibody titers over the long-term postoperative period. Additional examinations, such as FDG-PET/CT in combination with routine follow-up assessments, may be warranted to detect possible recurrent lesions when p53 antibody titers are elevated, even after a 5-year disease-free survival.

## CONCLUSIONS

We documented a case of pT1bN0 ESCC with lymphovascular invasion in which the patient had an elevated serum p53 antibody titer and developed recurrence in multiple mediastinal lymph nodes and pleural dissemination >5 years postoperatively. Even pT1bN0 diseases, in some cases, need careful long-term surveillance using imaging modalities.

## References

[1] Sugiyama M, Morita M, Yoshida R, et al. Patterns and time of recurrence after complete resection of esophageal cancer. Surg Today 2012; 42: 752–8. 22370963 10.1007/s00595-012-0133-9

[2] Umemura A, Akiyama Y, Iwaya T, et al. Super-late pulmonary recurrence after radical esophagectomy for esophageal squamous cell carcinoma. Int J Surg Case Rep 2020; 72: 166–71. 32535535 10.1016/j.ijscr.2020.05.068 PMC7299901

[3] Sekiguchi K, Matsutani T, Nomura T, et al. Pulmonary metastasectomy for esophageal basaloid squamous cell carcinoma component at 66 months after esophagectomy. Surg Case Rep 2020; 6: 199. 32757102 10.1186/s40792-020-00957-z PMC7406597

[4] Miyamoto H, Tamura T, Nishi S, et al. A case of late recurrence of esophageal cancer, 15 years after surgery. Gan To Kagaku Ryoho 2022; 49: 1570–2. (in Japanese) 36733138

[5] Hayashi T, Nishimaki T, Suzuki T, et al. Three cases of lymph node recurrence after a long disease-free interval of over 5 years following curative resection for thoracic esophageal carcinoma. Acta Med Biol (Niigata) 2000; 48: 107–11.

[6] Ota Y, Minamide J, Takata K, et al. A case of advanced esophageal cancer that has come back eight years after combined modality therapy. Gan To Kagaku Ryoho 2009; 36: 2442–4. (in Japanese) 20037450

[7] Yamamoto T, Tokunou K, Toshimitsu H, et al. A case of recurrent esophageal cancer successfully treated by combined therapies. Gan To Kagaku Ryoho 2010; 37: 2397–9. (in Japanese) 21224585

[8] Nakano A, Hoshino I, Kawahira H, et al. A case of metastasis within the gastric tube from esophageal squamous cell carcinoma which developed nine years after curative esophagectomy. Nihon Rinsho Geka Gakkai Zasshi 2011; 72: 2530–4. (in Japanese)

[9] Takahashi M, Hoshino I, Kano M, et al. A case of ovarian metastasis from esophageal squamous cell carcinoma that developed 5 years after curative esophagectomy. Nihon Rinsho Geka Gakkai Zasshi 2013; 74: 2761–5. (in Japanese)

[10] Mori O, Tomibayashi A, Eto S, et al. A case of esophageal cancer with superior mediastinal lymph nodes recurrence developed more than 9 years after radical resection. Nihon Rinsho Geka Gakkai Zasshi 2020; 81: 54–9. (in Japanese)

[11] Rice TW, Patil DT, Blackstone EH. 8th edition AJCC/UICC staging of esophagus and esophagogastric junction cancers: application to clinical practice. Ann Cardiothorac Surg 2017; 6: 119–30. 28447000 10.21037/acs.2017.03.14 PMC5387145

[12] Nakanoko T, Morita M, Nakashima Y, et al. Nationwide survey of the follow-up practices for patients with esophageal carcinoma after radical treatment: historical changes and future perspectives in Japan. Esophagus 2022; 19: 69–76. 34383154 10.1007/s10388-021-00869-3

[13] Hiyoshi Y, Yoshida N, Watanabe M, et al. Late recurrence after radical resection of esophageal cancer. World J Surg 2016; 40: 913–20. 26552914 10.1007/s00268-015-3334-8

[14] Zhu JF, Feng XY, Zhang XW, et al. Time distribution of recurrence risk of oesophageal squamous cell carcinoma with complete resection (R0) in a Chinese population. Eur J Cardiothorac Surg 2015; 48: 899–905. 25899517 10.1093/ejcts/ezv147

[15] Wang S, Chen X, Fan J, et al. Prognostic significance of lymphovascular invasion for thoracic esophageal squamous cell carcinoma. Ann Surg Oncol 2016; 23: 4101–9. 27436201 10.1245/s10434-016-5416-8

[16] Ohsawa M, Hamai Y, Emi M, et al. Recurrence and prognostic predictors in pathologic T1N0 esophageal squamous cell carcinoma treated with surgery alone. Surgery 2025; 178: 108863. 39419644 10.1016/j.surg.2024.09.019

[17] Huang Q, Luo K, Chen C, et al. Identification and validation of lymphovascular invasion as a prognostic and staging factor in node-negative esophageal squamous cell carcinoma. J Thorac Oncol 2016; 11: 583–92. 26792626 10.1016/j.jtho.2015.12.109

[18] Kikuchi E, Margulis V, Karakiewicz PI, et al. Lymphovascular invasion predicts clinical outcomes in patients with node-negative upper tract urothelial carcinoma. J Clin Oncol 2009; 27: 612–8. 19075275 10.1200/JCO.2008.17.2361 PMC2737380

[19] Udagawa T. Tumor dormancy of primary and secondary cancers. Acta Pathol Microbiol Scand Suppl 2008; 116: 615–28. 10.1111/j.1600-0463.2008.01077.x 18834406

[20] Endo H, Inoue M. Dormancy in cancer. Cancer Sci 2019; 110: 474–80. 30575231 10.1111/cas.13917 PMC6361606

[21] Takashi S, Satoshi Y, Akihiko O, et al. Clinical impact of preoperative serum p53 antibody titers in 1487 patients with surgically treated esophageal squamous cell carcinoma: a multi-institutional study. Esophagus 2021; 18: 65–71. 32715348 10.1007/s10388-020-00761-6

[22] Nakajima K, Takiguchi S, Mori M, et al. Peritherapeutic serum p53 antibody titers are predictors of survival in patients with esophageal squamous cell carcinoma undergoing neoadjuvant chemotherapy and surgery. World J Surg 2017; 41: 1566–74. 28108772 10.1007/s00268-017-3894-x

[23] Sørensen RB, Andersen RS, Svane IM, et al. CD8 T-cell responses against cyclin B1 in breast cancer patients with tumors overexpressing p53. Clin Cancer Res 2009; 15: 1543–9. 19223507 10.1158/1078-0432.CCR-08-1412

[24] Kawashima H, Takatori H, Suzuki K, et al. Tumor suppressor p53 inhibits systemic autoimmune diseases by inducing regulatory T cells. J Immunol 2013; 191: 3614–23. 24006461 10.4049/jimmunol.1300509

